# Phlegmonous Gastritis: A Case Report of Successful Early Antibiotic Treatment

**DOI:** 10.7759/cureus.13359

**Published:** 2021-02-15

**Authors:** Andrea DeCino, Jose Lisandro Gonzalez Martinez, Randy Wright

**Affiliations:** 1 Department of Internal Medicine, University of Texas Health Science Center at San Antonio, San Antonio, USA; 2 Department of Gastroenterology, University of Texas Health Science Center at San Antonio, San Antonio, USA

**Keywords:** gastritis, phlegmonous gastritis, suppurative gastritis, sepsis

## Abstract

Phlegmonous gastritis (PG) is a rare and serious bacterial infection of the gastric submucosa. Diagnosis is often delayed due to nonspecific symptoms, but if recognized early, PG may be treated successfully with medical therapy alone. We describe a case of a 47-year-old patient admitted with gastrointestinal symptoms and sepsis. He was found to have beta-hemolytic streptococcus bacteremia with a purulent gastric ulcer on endoscopic evaluation, consistent with the diagnosis of PG. Though surgical evaluation is often required in cases of PG, our patient quickly improved with parenteral antibiotic therapy. This case highlights an uncommon source of sepsis and demonstrates the success of antibiotic monotherapy with early recognition.

## Introduction

Phlegmonous gastritis (PG) is an uncommon, progressive, and frequently fatal bacterial infection of the gastric wall. With low incidence and nonspecific symptoms, diagnosis requires high clinical suspicion for further investigation. In delayed diagnosis, mortality can reach 42% [1]. No standardized therapy has emerged, and surgical intervention remains an important therapeutic modality. However, early initiation of medical therapy alone has been shown to be successful in a subset of patients [2]. We present a case of PG successfully treated with early antibiotic therapy. This article was previously presented as a meeting abstract at the 2019 ACG Annual Scientific Meeting on October 27, 2019.

## Case presentation

**Figure 1:**
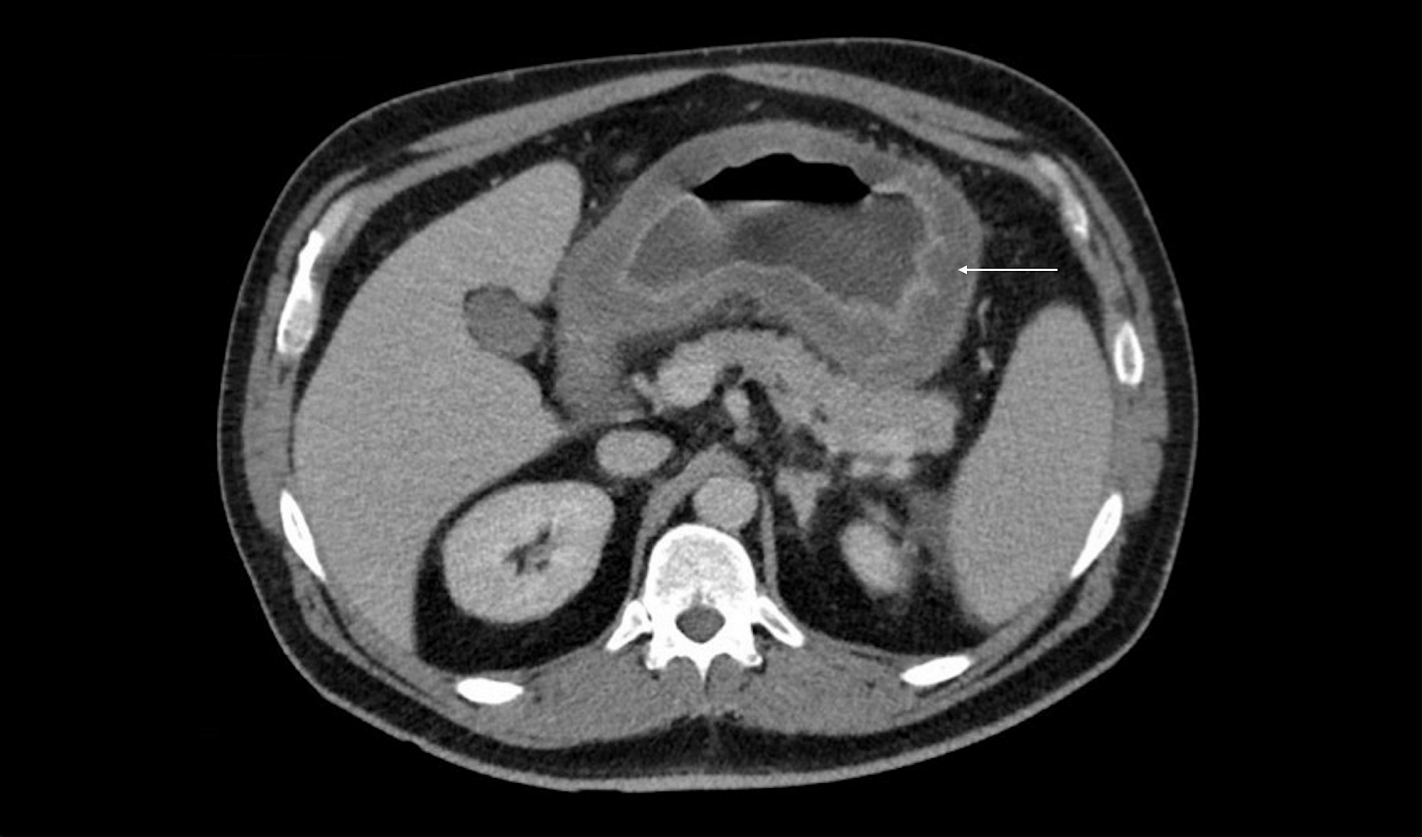
Contrast-enhanced abdominal CT demonstrating diffuse gastric wall thickening (up to 1.6 cm) with mucosal enhancement CT: computed tomography

A 47-year-old man presented with four days of subjective fevers, abdominal pain, and vomiting. Past medical history was remarkable for hypertension and uncontrolled type 2 diabetes mellitus (HbA1c 11.9%). There was no history of nonsteroidal anti-inflammatory drugs or alcohol use. No prior endoscopies were available for review. On admission, the patient was tachycardic to 122 beats/min. Physical examination was remarkable for epigastric tenderness without guarding or rebound tenderness. Admission laboratory studies revealed a leukocytosis of 30.3 K/mcL (normal: 3.4-10.4 K/mcL). Blood cultures were obtained, and the patient was started on piperacillin/tazobactam within five hours of presentation to the emergency department. Contrast-enhanced abdominal computed tomography (CT) revealed diffuse gastric wall thickening up to 1.6 cm with mucosal enhancement extending to the proximal duodenum (Figure 1).

**Figure 2:**
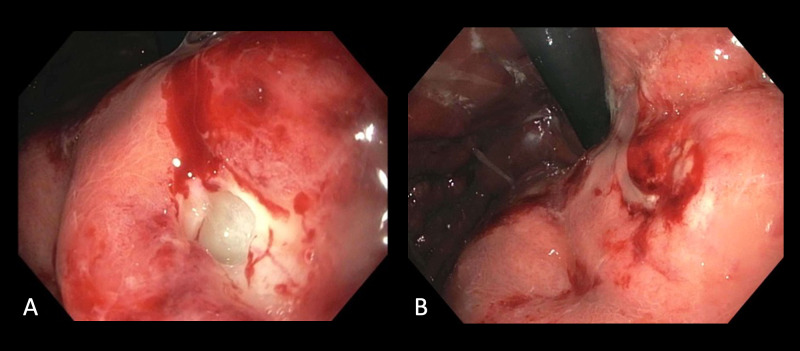
Endoscopic images of purulent drainage following deep biopsy of the ulcer in the lesser curvature of the gastric body

Esophagogastroduodenoscopy (EGD) redemonstrated gastric thickening with diffuse erythema and a 6-mm nonbleeding ulcer in the lesser curvature of the gastric body. Deep biopsy of the ulcer by cold forceps produced purulent drainage (Figures 2A, 2B). The duodenal bulb and second portion of the duodenum were normal.

Blood cultures returned positive for group A beta-hemolytic streptococcus and antibiotics were de-escalated to ampicillin/sulbactam. Endoscopic ultrasonography (EUS) was performed to evaluate for a component of gastric cancer and found a 1.2-cm diffusely thickened gastric wall, involving the superficial and deep mucosa.

**Figure 3:**
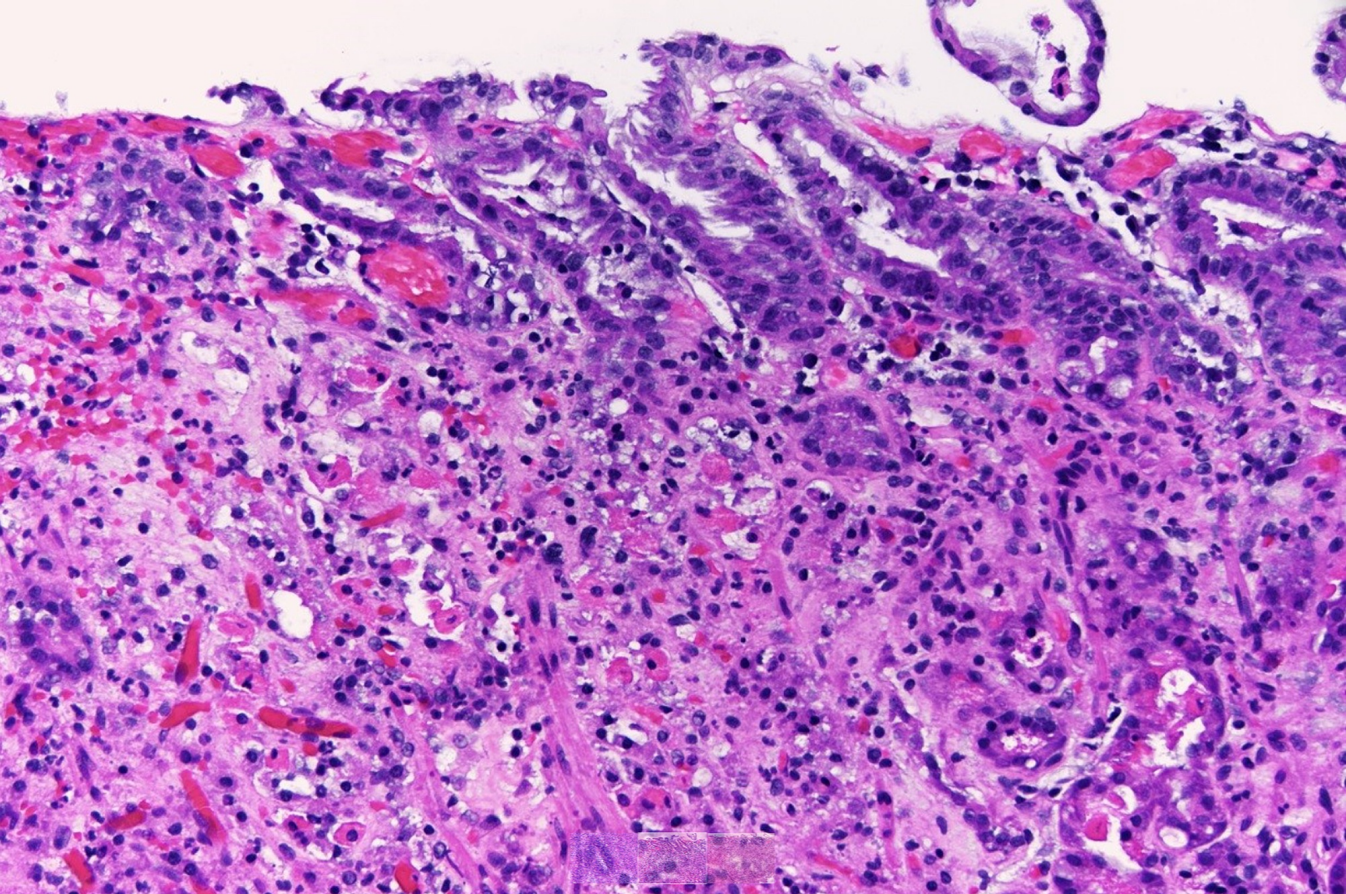
Hemoxylin and eosin stain at 20x magnification of deep gastric ulcer biopsy, consistent with erosions and full-thickness glandular degeneration with marked neutrophil infiltration

Biopsies from both EGD and EUS were consistent with gastritis and were negative for malignancy. Deep biopsies from the initial EGD demonstrated scattered neutrophilic micro-abscesses within superficial and deep gastric mucosa, with focal destruction of glandular epithelium (Figure 3). Endoscopic bacterial cultures were not obtained, and no organisms were identified on histopathology. The patient completed a 10-day course of parenteral antibiotics with a resolution of symptoms but did not follow up for repeat endoscopy.

## Discussion

PG is a rare clinical entity in which bacterial infection of the gastric submucosa can cause rapid fatality. Originally described in 160 AD by Galen and reported in the medical literature in 1862 by Cruveilhier, PG has maintained regular recognition as an important clinical entity since the 1920s [3-5]. Most information has been garnered through single case reports, and much is still unknown. A 2018 review of 25 cases found only four reports in the United States in the previous 10 years, with most literature coming from Japan and Korea [2].

Risk factors

Thought to be caused by either local or hematogenous spread of infection, PG can be classified under primary or secondary types [1,2,6,7]. Primary PG is often idiopathic in the setting of direct mucosal injury which can occur from chronic gastritis, peptic ulcer disease, post-endoscopic injury, and has even been reported after feeding tube placement [2,6,7]. Infection may involve the entire stomach or may be localized, most commonly in the antrum [2,3,8].

Immunosuppression is also recognized as a major risk factor. Chronic alcoholism is the most widely recognized, though diabetes mellitus, malnutrition, and malignancy have also been associated with the development of PG [2,3,6]. However, many patients have no obvious risk factors. Kim et al. found in 37 cases, 41% of patients were previously healthy with no predisposing risk factors identified [1]. 

Diagnosis

High clinical suspicion and early radiographic testing are important to identify PG, as clinical manifestations are nonspecific and up to 50% of diagnoses are made at autopsy [6]. Purulent emesis is considered pathognomonic, though rarely occurs in clinical practice [1]. Imaging can be supportive, for which EUS is superior to CT in assessing the extent of gastric wall thickness, though has not been routinely recommended [9]. Most patients also require direct visualization for evaluation of underlying malignancy. Endoscopic findings may include fibrinopurulent exudates lining the stomach, loss of rugae, edematous mucosa, and superficial ulcerations [6].

Infection most commonly involves the gastric submucosa, though may extend deeper in more severe cases [1,2]. In the absence of transmural infection, standard forceps biopsy does not sample sufficient submucosal tissue, and histopathology is of limited benefit. Snare biopsy specimens are of greater diagnostic value, and in cases of transmural involvement, histopathology reveals infiltration of neutrophils and plasma cells with intramural hemorrhage and necrosis [1,6,8,9].

Typical pathogens

*Streptococcus *spp. account for approximately 70% of cases, and up to one-third of infections are polymicrobial [1-3,7]. *Eischeria coli*, *Haemophilus influenza*, *Staphylococcus* spp., *Proteus*, and oral flora have also been implicated in the disease [1,3,7,9,10]. In our patient, the origin of infection remains unclear. The direct mucosal injury seems most likely, as the purulent ulcer seen on endoscopy may have served as a nidus of infection. Though the ulcer was not cultured and no organisms were found on multiple biopsies, we speculate that diagnostic yield was lowered due to our patient having received three days of parenteral antibiotics prior to sampling.

Treatment

Prior to antibiotic use, mortality was reported as high as 92% [1,3,6]. In the 1920s, the localized disease was primarily treated by surgical resection, while patients with diffuse disease underwent a procedure wherein multiple punctures of the stomach wall allowed for free drainage of purulent fluid [4].

More recently, mortality rates have ranged from 17% to 33%, though it remains unclear whether surgical or medical management is optimal [1,2,6]. In 36 cases reported from 1973 to 2003, there was a decreased mortality rate in early gastric resection when compared to medical management alone (20% vs 50%) [1]. Conversely, Iqbal et al. reviewed 25 cases from 2007 to 2017 and found an increase in both the number of patients successfully managed with medical therapy alone and an improved overall mortality rate of 12% [2]. This suggests that it is reasonable to defer surgical intervention in favor of trialing antibiotics, particularly in early diagnosis, and to reserve invasive treatment for patients who fail to improve or who develop complications [2]. Broad-spectrum empiric antibiotic therapy should be initiated, given high rates of polymicrobial infection and reports of multi-drug resistant pathogens [1,2].

There is no standard guidance regarding further management beyond clinical improvement. By week 4 following hospital discharge, several patients demonstrated healing on follow-up EGD [7,9,11]. In patients with persistent endoscopic abnormalities, the most common finding was atrophic gastritis [1,8]. Despite often normal findings on surveillance endoscopy, at least one follow-up EGD may be warranted to ensure healing and to exclude underlying malignancy.

## Conclusions

PG is a rare bacterial infection of the gastric submucosa that poses diagnostic and therapeutic challenges. While early diagnosis is critical to improving mortality, a significant portion of patients present with nonspecific symptoms without any known risk factors. PG should be considered in patients presenting with abdominal pain, vomiting, and infectious symptoms refractory to conventional treatment or when presenting critically ill, as in sepsis syndrome. Knowledge of the disease process and high clinical suspicion will lead to earlier initiation of broad-spectrum antibiotics and improved outcomes without the need for surgical intervention.
